# Temporal Discrimination: Mechanisms and Relevance to Adult-Onset Dystonia

**DOI:** 10.3389/fneur.2017.00625

**Published:** 2017-11-28

**Authors:** Antonella Conte, Eavan M. McGovern, Shruti Narasimham, Rebecca Beck, Owen Killian, Sean O’Riordan, Richard B. Reilly, Michael Hutchinson

**Affiliations:** ^1^Department of Neurology and Psychiatry, Sapienza, University of Rome, Rome, Italy; ^2^IRCCS Neuromed, Pozzilli, Isernia, Italy; ^3^Department of Neurology, St Vincent’s University Hospital Dublin, Dublin, Ireland; ^4^School of Medicine and Medical Science, University College Dublin, Dublin, Ireland; ^5^Trinity Centre for Bioengineering, Trinity College, The University of Dublin, Dublin, Ireland; ^6^School of Medicine, Trinity College, The University of Dublin, Dublin, Ireland; ^7^School of Engineering, Trinity College, The University of Dublin, Dublin, Ireland

**Keywords:** temporal discrimination threshold, cervical dystonia, blepharospasm, adult-onset focal dystonia, superior colliculus, endophenotype

## Abstract

Temporal discrimination is the ability to determine that two sequential sensory stimuli are separated in time. For any individual, the temporal discrimination threshold (TDT) is the minimum interval at which paired sequential stimuli are perceived as being asynchronous; this can be assessed, with high test–retest and inter-rater reliability, using a simple psychophysical test. Temporal discrimination is disordered in a number of basal ganglia diseases including adult-onset dystonia, of which the two most common phenotypes are cervical dystonia and blepharospasm. The causes of adult-onset focal dystonia are unknown; genetic, epigenetic, and environmental factors are relevant. Abnormal TDTs in adult-onset dystonia are associated with structural and neurophysiological changes considered to reflect defective inhibitory interneuronal processing within a network which includes the superior colliculus, basal ganglia, and primary somatosensory cortex. It is hypothesized that abnormal temporal discrimination is a mediational endophenotype and, when present in unaffected relatives of patients with adult-onset dystonia, indicates non-manifesting gene carriage. Using the mediational endophenotype concept, etiological factors in adult-onset dystonia may be examined including (i) the role of environmental exposures in disease penetrance and expression; (ii) sexual dimorphism in sex ratios at age of onset; (iii) the pathogenesis of non-motor symptoms of adult-onset dystonia; and (iv) subcortical mechanisms in disease pathogenesis.

The purpose of this review is to present and analyze, from clinical, neurophysiological, and neuroimaging studies, the evidence as to the biological basis of temporal discrimination and the role of abnormal temporal discrimination in understanding the pathogenesis of adult-onset focal dystonia.

## The Temporal Discrimination Threshold (TDT)

The TDT (sometimes referred to as “simultaneity judgment” when using multi-modal stimuli) is defined as the shortest interval at which two sequential sensory stimuli are perceived as being asynchronous (1). The sequential sensory stimuli may be visual, tactile, or auditory; paired tactile stimuli have been used most commonly, some studies have used multi-modal stimuli (visual followed by tactile). Proprioceptive TDT (temporal discrimination of movement threshold) can be assessed as the shortest interval at which two electrically elicited paired muscle twitches are perceived as asynchronous (2–4). Although the TDT is one of the many ways to evaluate temporal processing of sensory information, the neurophysiological basis of temporal discrimination has distinct attributes, not shared with these other measures. The rapid automatic detection and processing of temporal change in the sensory environment, through temporal discrimination, is a mechanism which enables the basal ganglia to select an immediate, protective, motor programme.

The TDT is relatively easy to assess in the laboratory or even, by using a portable headset, in the participant’s home (5). The stimulus protocol used to measure the TDT is a major determinant of performance; detailed descriptions of laboratory methods are provided in the Supplementary Material. Technical variables, which must be controlled, in order to obtain reproducible results, include stimulus mode and intensity, presentation sequence. Participant characteristics which can markedly affect temporal discrimination include age, sex, medication, and comorbid (neurological) disorders.

### Participant Variables Affecting Temporal Discrimination

#### Age-Related Effects

An early study of 80 healthy volunteers aged from 18 to 82 years found that TDT increased only in subjects older than 65 years (6). Other investigators found, in 100 healthy volunteers aged 18–79 years, an increase in the TDT by 0.66 ms for every year increase in age (7). Age-related increase in TDT is probably multifactorial. Proposed causative factors include changes in inhibitory GABA-interneuron activity, and iron deposition in the brain areas involved in testing (7). Our experience in healthy participants older than 65 years is that, because of marked increase in variance in the TDT, it is extremely difficult to determine, with adequate sensitivity and specificity, normal and abnormal TDTs in this age group using *Z*-scores.

#### Sex

An intriguing interaction of the relationship between age and sex, so far unreported by others, has been described (8, 9). Young women (less than 40 years of age) performed better in temporal discrimination than men. However, age-related decline in the TDT was three times faster in women; thus men, with age, after 45 years, had increasingly faster temporal discrimination (relative to women). Because of the age and sex-related effects on temporal discrimination, well-defined normal TDT values require the acquisition of TDTs in 150–200 control participants to cover the age range 20–65 years in both sexes (10).

#### Participant Comorbidities

There are numerous comorbid disorders, which need to be screened for prior to study, which may affect TDT testing. For visual TDTs, corrected visual acuity needs to be assessed and patients with any condition resulting in loss of visual acuity, need to be excluded. For tactile TDT testing, impaired superficial sensation needs to be enquired about and examined. Also excluded are participants with a history of a neurological disorder or medication known to affect the basal ganglia. All participants should be screened for cognitive impairment using the Montreal Cognitive Assessment; cognitive impairment will affect the ability to understand and participate in the study.

#### Superior Temporal Discrimination: The Special Case of Musicians

Although intuitively plausible, there had been no evidence, until recently, that intensive training from an early age has an effect on the efficiency of temporal discrimination. Musicians perform better than non-musicians in visual timing tasks including rhythm perception and duration discrimination (11); however, the temporal discrimination task does not relate to rhythm perception or temporal duration. In a recent study, TDTs were measured in 20 healthy professional musicians and 94 healthy non-musicians (12). Healthy musicians had faster TDTs than healthy non-musicians at all ages. Healthy musicians also exhibited less age-related decline in temporal discrimination than non-musicians, suggesting some protective effect associated with playing an instrument. This finding is supported by a study which showed that a moderate amount (4–14 years) of music training early in life was associated with faster neural timing in response to speech later in life, even long after training had stopped (13).

### What Is Not Temporal Discrimination: Other Measures of Temporal Processing

Sub-second temporal processing of sensory information has been studied by several methodological approaches including temporal order judgment (TOJ), frequency discrimination task, time estimation tasks, and interval discrimination tasks (14–16). These various tasks differ from TDT in the neural circuits activated during the experimental procedure. In TOJ, participants receive two stimuli with a certain stimulus onset asynchrony and judge which stimulus is presented first. The TOJ paradigm has disclosed various psychophysical phenomena; for example, the observation that crossing the arms increases the rate of tactile TOJ misreporting (17) suggests that the brain processes hand spatial locations before temporally ordering tactile signals (18).

Frequency discrimination and interval discrimination usually involve time interval comparison. Intervals elapsing between the two stimuli vary in the hundred milliseconds range, a procedure implying that the structures for encoding differ from those for temporal discrimination, which encompasses tens of milliseconds (19). Unlike the TDT, these tasks require higher-order abilities such as attention and working memory (frequency discrimination and time estimation tasks) (20). Conversely, the TDT seems to be a perceptive threshold uninfluenced by memory formation (1, 21) and at the interval used, in the tens of milliseconds range, is beyond cognitive control (22).

## The Neuroanatomy of Temporal Discrimination

Evidence, from lesional and neurophysiological studies, suggests that TDT involves the time-locked activation of both subcortical and cortical neural networks. Which neural structures primarily determine temporal discrimination is still open to debate; does temporal discrimination require cortical activation or is it purely dependent on basal ganglia–brainstem–cerebellar integrity?

### The Subcortical Network

The role of the basal ganglia in temporal processing has been well known for decades. In an fMRI study in healthy subjects, Pastor and colleagues initially showed that, as well as primary somatosensory cortex and cerebellum, other areas, specifically active during temporal processing, included the pre-supplementary motor area (pre-SMA) and putamen (23). In a later paper, the same research group using fMRI, demonstrated in a temporal discrimination task that, only when participants were perceptually certain that either one stimulus was, or two stimuli were, perceived, this state of certainty was uniquely associated with putaminal activation (24). Rao and colleagues, in an fMRI study comparing the duration of two tones (not a temporal discrimination task) demonstrated that the putamen and caudate were involved early in a temporal processing task (25); a similar finding was reported in Ref. (26).

It is postulated that temporal discrimination is a measure of the acuity of an alerting circuit that signals the detection of biologically salient events (event or emergency) in order to modify on-going behavior (freeze or escape). Midbrain dopaminergic neurons produce short-latency responses to biologically salient events (27–30). A salient environmental sensory event (tactile or visual), is detected by the superior colliculus as environmental change. In response to a visual stimulus, most of the neurons in the superior colliculus exhibit transient “ON” responses within 50 ms of the stimulus onset (31). With a persistent visual stimulus, most of these cells enter a “PAUSE” phase and then only discharge again when the visual stimulus is switched off. The superior colliculus, through the sequence “ON–PAUSE–OFF” detects salient environmental changes and sends priority signals to the substantia nigra pars compacta and the intralaminar nucleus of the thalamus, thus exciting bursts in the striatal cholinergic interneurons, which activate the cascade of events ultimately selecting behavior appropriate to the environmental changes (32, 33). Studies in mice have shown that, with blocking of both GABAa and GABAb receptors in the superior colliculus, there is excessive and prolonged burst activity in both the “ON” and “OFF” phases with loss or attenuation of the normal “PAUSE” phase (31). This prolonged burst activity blunts the offsets when a visual stimulus is presented momentarily and thus the intervals between sequential visual stimuli at which they may be detected is prolonged, leading to an abnormal TDT. For this reason, it is considered that an abnormal TDT is a marker of defective inhibition within the superior colliculus or arising from substantia nigra pars compacta (34). Further strengthening the idea that these subcortical structures integrate temporal information comes from the altered TDT values reported in patients with various basal ganglia disorders (1, 35–39).

### The Cortex and Temporal Discrimination

The pre-SMA probably plays a role in focusing attention on the discriminative task but is not determinant in encoding TDT values (40). Temporal discrimination testing following repetitive transcranial magnetic stimulation (rTMS)-induced modulation of the pre-SMA found that TDT values were not modified but the number of errors in the catch trials was increased (40). Whatever the underlying mechanism, convincing research confirms that TDT processing also involves a cortical component. Several lines of evidence show that the primary somatosensory cortex refines TDT-related sensory information. Experiments conducted in our laboratory have shown that in healthy subjects S1 rTMS, a technique that induces changes in the stimulated cortical activity lasting about 30 min, modifies TDT values (40) as well as the somatosensory evoked potential (SEP) N20 component. Investigating whether S1 rTMS-induced changes in TDT relate to neural processing in S1, Rocchi et al. (41) found a correlation between TDT, SEP recovery cycles, and S1-high-frequency oscillations (HFOs). S1-HFOs are thought to represent postsynaptic activity in S1 inhibitory interneurons (42). Based on the correlation between the baseline and post-rTMS Sl-HFO, TDT values, and the degree of SEP recovery cycle inhibition, Rocchi and colleagues suggested that the three variables share common mechanisms and that the inhibitory circuits in S1 sharpen the distinction between potentially overlapping excitatory inputs between the first and second afferent volley (stimulus) in temporal discrimination testing (41). Investigating the cortical role in mediating tactile TDT in dystonic patients, Antelmi et al. (43) reported that increased TDT values were associated with reduced suppression of cortical and subcortical paired-pulse SEPs as well as with a smaller area of the HFO early component. Overall, these findings pointed to a reduced activity in dystonic patients of the inhibitory interneurons within the primary somatosensory cortex.

#### A Synthesis

**Figure 1:**
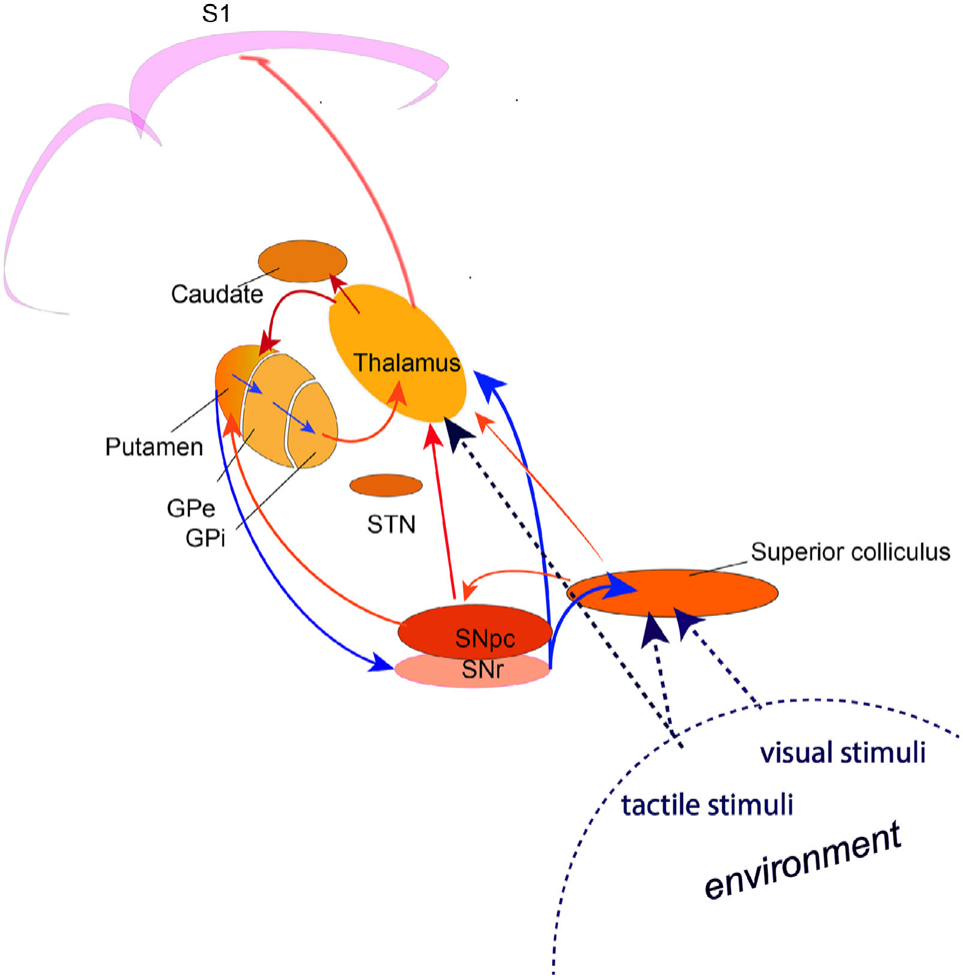
A circuit diagram illustrating the network involved in the process of temporal discrimination. Sensory stimuli (visual, tactile, auditory) entering the superior colliculus caused by external environmental change are processed through a feed forward pathway to the intralaminar nuclei of the thalamus and the substantia nigra pars compacta. Feed forward through the putamen and the direct and indirect pathways through the basal ganglia, results in reduced inhibition from the substantia nigra pars reticulata, allowing activation of the “GO” pathway and action selection (saccade and/or head turn) for emergency activity to inspect the source of the stimulus (event/environmental change) or to defend against it. (The broken lines indicate the multi-modal sensory inputs, which are processed through the superior colliculus. Blue arrows indicate inhibition; red arrows indicate excitation).

Accumulating evidence indicates that the TDT task relates to the detection of novel, salient environmental change involving a brainstem–cerebellar–basal ganglia neuronal network with the superior colliculus as a principal cross-modal sensory input node. A comprehensive hypothetical model for the neural circuits involved in normal temporal discrimination requires basal ganglia functional integrity in a network that integrates incoming sensory information from the superior colliculus, thalamic nuclei and cerebellum and selects salient events for on-going behavior through the dopamine-mediated alerting system (Figure 1). Temporal sensory inflow arriving at S1 cortex then sharpens the perceived threshold through inhibitory cortical interneuron activity.

## Temporal Discrimination and Adult-Onset Focal Dystonia

Adult-onset idiopathic isolated focal dystonia (AOIFD/adult-onset focal dystonia) is an uncommon movement disorder of unknown cause and pathogenesis (44). It presents clinically as one of different phenotypes: cervical dystonia, blepharospasm, oromandibular dystonia, focal hand dystonia, spasmodic dysphonia, musician’s dystonia (and other task-specific dystonias). The most common phenotypes are cervical dystonia and blepharospasm; cervical dystonia is more common in Northern Europe, whereas blepharospasm is more prevalent in Southern Europe. There are intriguing associations between the mean age of onset of adult-onset dystonia and sex ratios; there is a male predominance in phenotypes with age of onset under 40 years of age (particularly in focal hand dystonia and musician’s dystonia), but an increasing female predominance with increasing age of onset after the age of 40 (particularly in blepharospasm and spasmodic dysphonia) (45, 46). Most patients with adult-onset dystonia are sporadic, with no other affected family member, however, with investigation and further enquiry, 25% have at least one other affected relative, often with a different phenotype. It is considered that adult-onset dystonia is an autosomal dominant disorder with markedly reduced penetrance of 10–12% (47, 48).

Current concepts concerning AOIFD are: (1) AOIFD is an autosomal dominant disorder with markedly low penetrance (which explains why most cases are sporadic). (2) The different phenotypes are not caused by different genetic mutations; discordant phenotypes are seen in 50% of affected proband-relative pairs (e.g., blepharospasm in a brother and cervical dystonia in his sister) and are also seen in multiplex families (49). (3) Environmental factors determine both disease penetrance and expression (50, 51). (4) Both age at onset and sex determines (in part) the phenotype (46). Thus, environmental exposure history, age at onset, and sex are separate non-genetic and epigenetic factors determining disease penetrance and expression in an individual carrying susceptibility gene(s) for AOIFD. Most research studies of environmental effects in AOIFD involve cervical dystonia or blepharospasm patients because of their relatively greater prevalence.

### Temporal Discrimination in AOIFD

Abnormal temporal discrimination in AOIFD was initially described in 2001 (52) and confirmed from multiple centers since (53–58). It is important to repeat that temporal discrimination varies physiologically according to sex and age and robust age- and sex-related control values need to be established in any one laboratory. The prevalence of abnormal TDTs in AOIFD varies by phenotype being most sensitive and specific in cervical dystonia (59, 60). Abnormal TDTs demonstrate autosomal dominant transmission in unaffected first-degree relatives of patients with sporadic and familial cervical dystonia (10, 59, 61) with variable age- and sex-related penetrance (10) (Table 1).

**Table 1:** This table summarizes the research findings, described fully in the text, in relation to the endophenotype, abnormal temporal discrimination, and the phenotype, adult-onset dystonia.

Measure/variable	Endophenotype: abnormal temporal discrimination	Phenotype: adult-onset dystonia
Inheritance	Autosomal dominant	Autosomal dominant
Penetrance	100% in women by 48 years 40% in men by 40 years	10–15% penetrant
Age and sex interaction	Female:male ratio constant after 40 years	Increasing female: male sex ratio with increasing age of onset
Environmental exposures and expression	No effect on penetrance or expression	Important: determines both penetrance and expression
Diagnostic test	Simple psychophysical test high inter-rater reliability high test–retest reliability	None: expert opinion alone
Associated structural abnormality	Putaminal hypertrophy in carriers	Putaminal hypertrophy in:blepharospasm^a^ musician’s dystonia^b^
Botulinum toxin	No effect	Improves motor phenotype
Deep brain stimulation	No effect	Improves motor phenotype
Pathomechanisms	Proven superior colliculus-basal ganglia mechanisms	Unknown: not obvious from phenotype
Non-motor syndrome present	Not examined	High prevalence; up to 70%
Secondary endophenotypes	None	Numerous

*Given that cervical dystonia is the most common phenotype of adult-onset dystonia, most of the relevant studies relate to patients with cervical dystonia and their unaffected first-degree relatives with and without abnormal temporal discrimination. The ^a^ refers to a report of putaminal hypertrophy in patients with blepharospasm (64); the ^b^ refers to a paper on putaminal hypertrophy in patients with musician’s dystonia (63)*.

### Abnormal Temporal Discrimination: A Mediational Endophenotype in AOIFD

We consider that an abnormal TDT is a mediational endophenotype of AOIFD. This implies that the endophenotype and the disease are both caused by a genetic disorder and that the pathway from gene to disease passes through the endophenotype; one cannot acquire the disease without first having the endophenotype (62). A mediational endophenotype reflects disease susceptibility, is not altered by disease severity, is closer to genetic mechanisms of expression, and is more penetrant than the phenotype.

### The Putamen, Abnormal Temporal Discrimination, and Adult-Onset Focal Dystonia

Although many publications report cortical abnormalities in AOIFD, these abnormalities may be secondary, adaptive changes in response to the motor manifestation of dystonia. One structural abnormality, coherent with the endophenotype, abnormal temporal discrimination, is alteration in the size of the putamen. Unaffected relatives (of cervical dystonia patients) with abnormal TDTs, when compared to relatives with normal TDTs have: (1) larger putaminal volumes by voxel-based morphometry (59), (2) reduced putaminal activity when performing a temporal discrimination task during an fMRI study (10), and (3) reduced responses in the superior colliculus, by fMRI to a looming visual stimulus (Hutchinson, unpublished research).

### Putamen, Musician’s Dystonia, and Temporal Discrimination

Putaminal enlargement is found in musician’s dystonia patients; the degree of putaminal enlargement correlates with the degree of keystroke irregularity (as a marker of severity of MD) (63). Putaminal enlargement has also been reported in other forms of adult-onset dystonia [blepharospasm (64)]. There are significant correlations between abnormal temporal discrimination and putaminal function and structure in sporadic laryngeal dystonia (65). The putamen is involved early in a temporal discrimination task and, by fMRI, is activated when paired stimuli are perceived distinctly as single or double (24). Thus, putaminal enlargement appears to reflect dysfunction both in adult-onset focal dystonia and in temporal discrimination.

Diffusion tensor imaging (DTI) is a robust method used to analyze structural and functional connectivity changes and characterize microstructural white matter changes in multiple regions of the brain in dystonia patients. Unfortunately, no studies have examined functional connectivity in relation to temporal discrimination in adult-onset focal dystonia. DTI research in patients and unaffected relatives with and without abnormal temporal discrimination is warranted.

The observations that abnormal TDTs in dystonic patients do not correlate with disease severity (56) are independent from the body part affected by dystonia and do not improve after botulinum toxin injection (57) or deep brain stimulation (66) suggest that increased TDT are not directly linked to the motor manifestations of dystonia. In further confirmation of this hypothesis, a recent study showed that in patients with a prodromal form of blepharospasm (increased blinking) TDT abnormalities were present before the development of blepharospasm (67).

Dystonia has been considered a network disorder in which different neurophysiological mechanisms have been consistently reported including reduced inhibitory activity, altered sensorimotor integration, and abnormally increased plasticity mechanisms. Abnormal temporal discrimination in AOIFD possibly reflects abnormal inhibitory interneuronal activity at different levels of the CNS, including subcortical structures (basal ganglia and superior colliculus) and S1 cortex (34, 43, 68). In conclusion, TDT abnormalities in dystonia possibly reflect a defective midbrain network—with superior colliculus as a central node—which signals salient changes in sensory events and a defective sharpening of perceived sensory stimuli at the cortical level; both of these processes are mediated by GABA-inhibitory interneuronal activity.

Since cortical plasticity mechanisms rely on a dynamic balance between excitatory and inhibitory interneurons (69), it is conceivable that altered inhibitory interneuron activity may concur to give rise to other pathophysiological mechanisms reported in dystonia, such as aberrant cortical plasticity mechanisms (70).

## Temporal Discrimination: Solving Problems in Adult-Onset Dystonia

**Figure 2:**
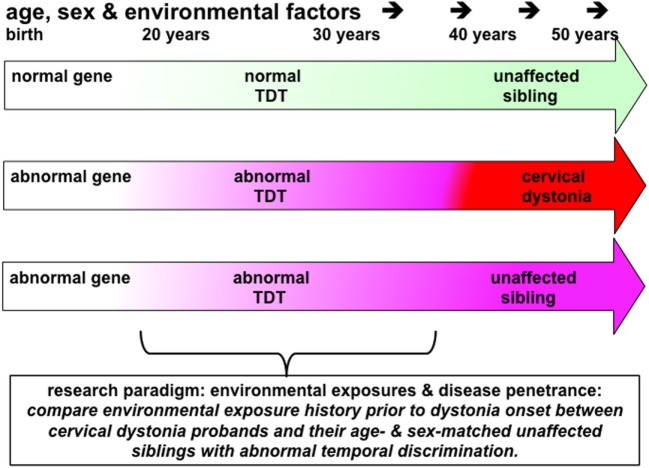
This figure illustrates the principles underling a study to investigate the effects of environmental exposures in disease penetrance in cervical dystonia (or blepharospasm). Probands, patients with cervical dystonia (red arrow), would be matched with same sex and similarly aged (−5/+10 years) unaffected siblings (pink arrow) who have abnormal temporal discrimination thresholds (TDTs). Environmental exposures in each proband–sibling matched pair would be assessed by completing a standardized environmental history questionnaire. Power calculations suggest that 60 such pairs would be sufficient to detect an 8% difference in exposure rates at an alpha of 5%. This study protocol could be considered to be a comparison of environmental histories between manifesting and non-manifesting gene carriers. Given the 100% penetrance, of abnormal TDTs in women after the age of 48 years, compared to the reduced penetrance in men (40% after 25 years of age), successful recruitment to such a study would require recruitment of predominantly women, aged 45–65 years of age, with their similarly aged sisters. It is likely that such a study would require collaborative work between three or more centers.

By using TDT testing in patients and their unaffected relatives, we can, by hypothesis-based research protocols, answer a number of fundamental questions on the pathogenesis of adult-onset focal dystonia (Table 1 and Figure 2).

### Environmental Factors in Cervical Dystonia and Blepharospasm

The profoundly low penetrance of adult-onset dystonia (10–15%) indicates that there are a number of non-genetic factors, which affect both disease development and its expression as a particular phenotype. Overuse, as in musician’s dystonia and writer’s cramp, is well recognized (71). However, in cervical dystonia, there are no obvious environmental factors and no evidence of overuse as a mechanism. Comparing environmental histories in a cohort of cervical dystonia probands and their unaffected siblings can be used to determine environmental exposures which increase the risk of a disease and which may protect against it. By hypothesizing that siblings with abnormal temporal discrimination are non-manifesting gene carriers, one can compare environmental histories between manifesting and non-manifesting gene carriers (Figure 2).

### Pathomechanisms of Adult-Onset Focal Dystonia

Neurophysiological or imaging studies comparing unaffected relatives with normal temporal discrimination and unaffected relatives with abnormal temporal discrimination are powerful tools to assess pathomechanisms in dystonia. They have the advantage that secondary changes in the brain due to disease expression (secondary endophenotypes), such as remodeling in the cortex, do not complicate matters.

### The Non-Motor Syndrome of Adult-Onset Focal Dystonia

Although adult-onset dystonia presents as a motor disorder, non-motor symptoms (comprising the “non-motor syndrome”) include sensory, neuropsychiatric, and sleep disorders (72); these are increasingly recognized and importantly they precede the motor symptoms by many years (73). Examination of the prevalence of non-motor symptoms in unaffected, age and sex-matched, first-degree relatives with and without abnormal temporal discrimination would be a useful experiment. If psychiatric symptoms are more prevalent in relatives with abnormal temporal discrimination, then it could be argued that they are part of the premotor syndrome and indicate subclinical meso-limbic disease penetrance, prior to motor manifestation.

### Explaining the Age-Related Sexual Dimorphism at Age of Onset of Adult-Onset Focal Dystonia

Temporal discrimination in unaffected relatives of cervical dystonia patients and sex ratios in adult-onset dystonia phenotypes show similar patterns of age-related sexual dimorphism. AOIFD with onset below the age of 40 years is male predominant (in focal hand dystonia and musician’s dystonia). After 40–45 years of age and, importantly, with increasing age there is a steady linear increase in the proportion of women affected in the craniocervical phenotypes. The male:female sex ratio (proportion of men) in AOIFD decreases with increasing mean age at onset; this association is highly significant; mean age of onset accounts for almost 60% of the variance in the proportion of men (46). This age-related sexual dimorphism in sex ratios at age of onset of AOIFD is unexplained. Temporal discrimination also shows age-related sexual dimorphism (46). Such age-related sexual dimorphism in temporal discrimination and adult-onset focal dystonia may reflect common underlying mechanisms. Cerebral GABA levels have been reported to show similar age-related sexual dimorphism in healthy participants (74) and may be the mechanism underlying the observed age-related sexual dimorphism in temporal discrimination and the sex ratios in adult-onset isolated focal dystonia.

## Conclusion

Consistent evidence shows that TDT testing in dystonia has shed light into the pathophysiological mechanisms of dystonia. Future studies using the TDT in carefully constructed clinical research protocols may address important questions in relation to both disease penetrance and disease expression in adult-onset dystonia.

## Author Contributions

Substantial contributions to the conception or design of the work; or the acquisition, analysis, or interpretation of data for the work: AC, MH, EM, SN, RB, OK, SO, and RR. Drafting the work or revising it critically for important intellectual content: AC, EM, SN, RB, OK, SO, RR, and MH. Final approval of the version to be published: AC, EM, SN, RB, OK, SO, RR, and MH. Agreement to be accountable for all aspects of the work in ensuring that questions related to the accuracy or integrity of any part of the work are appropriately investigated and resolved: AC and MH.

## Conflict of Interest Statement

The authors declare that the research was conducted in the absence of any commercial or financial relationships that could be construed as a potential conflict of interest.
