# Supplementary material for: Temporal Discrimination: Mechanisms and Relevance to Adult-Onset Dystonia
Source: Front Neurol. 2017 Nov 28;8:625. doi: 10.3389/fneur.2017.00625 (PMC5712317; doi:10.3389/fneur.2017.00625)
Supplement: Supplementary file 1 [file Data_Sheet_1.docx]

**Supplementary material**

***Measuring and analysing temporal discrimination:***

***Laboratory techniques:***

**The tactile TDT** is measured by delivering paired electrical stimuli over the skin on a body region: test values vary little between different body regions (e.g. face, neck, finger, and toe) or body sides (Conte *et al.,* 2010; Lee *et al.,* 2005, Lyoo *et al.,* 2007; Scontrini *et al.,* 2009). The intensity of electrical stimulation needed for testing tactile TDT is about 1.2 to 2 times the threshold of sensory perception, well below the pain threshold, and results in reproducible TDTs (Ramos *et al.,* 2016, Conte *et al.,* 2016b).

**For visual TDT testing** two LED lights with a luminance of 90cd/m2 are positioned seven degrees into the visual fields (alternating left and right) of the participant and horizontally orientated on the table in front of the subject. Participants are asked to look at a focal point in the midline and to try not to look directly at the flashing lights. An additional small amount of background luminance is used to enable the operator to see just enough in the dark environment to run the test. Pairs of lights are synchronised initially and thereafter are progressively separated in 5 ms steps every 5 seconds (Staircase Method). When the participant reports that pairs of lights are flashing asynchronously on three consecutive occasions, the first of these was taken as the visual TDT. The median of four trials on each side is used for each subject in order to allow for practice effect, and these two results (one from each side) are averaged to obtain a summary visual TDT score (in milliseconds)(Beck et al., 2018).

***Stimulus protocol:*** The stimulus protocol used to measure the TDT is a major determinant of performance. The sequence of inter-stimulus intervals (ISIs) between the first and second of the paired stimuli can be 1): Stepwise (staircase) and either a): *Increased* from a sub-threshold ISI until the subject reports the perception of two stimuli or, b): *Decreased,* starting from clearly separated ISI, until the participant reports one stimulus. An alternative is 2): Randomised presentation, with stimuli presented at randomly variable ISIs, and fitting data on a curve (Pastor *et al.,* 2004; Rocchi *et al.,* 2016; McGovern *et al.,* 2017). There is a significant relationship between the TDT values obtained with the two techniques (randomised versus staircase) confirming the robustness of both approaches (McGovern *et al.,* 2017). However, the staircase method yields lower TDT values than the randomised method, thus healthy control participant results are method-specific and should be uniformly applied in any one study. Both staircase and randomised methods seem unbiased by potential learning effects, provided that subjects are familiar with the procedure before formal assessment. The observation that TDT values remained unvaried across sessions in multiple session studies (Conte *et al.,* 2016; Ramos *et al.,* 2016) further indicates that the technique has fair-to-good reproducibility. Inter-rater reliability in experienced research staff is high (Kimmich *et al.,* 2014).

***Tabletop and Headset methods****:*

Most temporal discrimination testing is performed in hospital or university clinical neurophysiology laboratories; the equipment needed is relatively simple and well described in a number of reports. Recruitment of members of large, geographically dispersed, kindreds for research studies is challenging; a headset apparatus for visual TDT testing has recently been shown to be a reliable instrument for data acquisition (Molloy et al., 2014). Using the headset, the researcher can now travel to the family member, promoting recruitment of affected patients or unaffected relatives to clinical studies.

***Statistical analysis of participant’s responses:***

In most studies the TDT is taken as the shortest ISI at which a participant reports, on three occasions, the perception of asynchrony (two separate stimuli); a single value, in milliseconds, is taken as the TDT. An abnormal TDT is usually defined as being a Z-score ≥ 2.5 from the mean TDT, established from a cohort of age- & sex-matched healthy control participants. More recently two laboratories have suggested psychometric analytical techniques and using a non-parametric bootstrapping method to fit 95% confidence intervals upon individual participant’s data, to determine the (i) Point of Subjective Equality (PSE) and (ii) Just Noticeable Difference (JND) (Rocchi *et al.,* 2016; Butler *et al.,* 2015). The PSE value represents a more sensitive measure of temporal discrimination than the standard single TDT measure; it is of particular use in demonstrating differences when comparing relatively small numbers of control and clinical groups. The PSE & JND values have been robustly tested and may prove to be a more reliable means of detecting subtle differences within and between participant cohorts than a single TDT value.

***References:***

Beck RB, McGovern EM, Butler JS, Birsanu D, Quinlivan B, Beiser I, et al. Measurement & Analysis of the Temporal Discrimination Threshold Applied to Cervical Dystonia. J Vis Exp (2018) (in press).

Butler JS, Molloy A, Williams L, Kimmich O, Quinlivan B, O’Riordan S, et al. Non-parametric bootstrapping method for measuring the temporal discriminationthreshold for movement disorders. J Neural Eng (2015) 12(4):046026. doi:10.1088/1741-2560/12/4/046026.

Conte A, Modugno N, Lena F, Dispenza S, Gandolfi B, Iezzi E, et al. Subthalamic nucleus stimulation and somatosensory temporal discrimination in Parkinson’s disease. Brain (2010) 133:2656–63. doi:10.1093/brain/awq191.

Conte A, Belvisi D, Manzo N, Bologna M, Barone F, Tartaglia M, et al. Understanding the link between somatosensory temporal discrimination and movement execution in healthy subjects. Physiol Rep (2016) 4:e12899. doi:10.14814/phy2.12899.

Kimmich O, Molloy A, Whelan R, Williams L, Bradley D, Balsters J, et al. Temporal discrimination, a cervical dystonia endophenotype: penetrance and functional correlates. Mov Disord (2014) 29:804–11. doi:10.1002/mds.25822.

Lee MS, Kim HS, Lyoo CH. “Off” gait freezing and temporal discrimination threshold in patients with Parkinson disease. Neurology (2005) 64:670–4. doi:10.1212/01.WNL.0000151961.14861.BA.

Lyoo CH, Lee SY, Song TJ, Lee MS. Abnormal temporal discrimination threshold in patients with multiple system atrophy. Mov Disord (2007) 22:556–9.doi:10.1002/mds.21111

McGovern EM, Butler JS, Beiser I, Williams L, Quinlivan B, Narasiham S, et al. A comparison of stimulus presentation methods in temporal discrimination testing. Physiol Meas (2017) 38:57–64. doi:10.1088/1361-6579/38/2/N57.

Molloy A, Kimmich O, Williams L, Quinlivan B, Dabacan A, Fanning A, et al. A headset method for measuring the visual temporal discrimination threshold in cervical dystonia. Tremor Other Hyperkinet Mov (N Y) (2014) 4:249. doi:10.7916/D8TD9VF6

Pastor MA, Day BL, Macaluso E, Friston KJ, Frackowiak RS. The functional neuroanatomy of temporal discrimination. J Neurosci (2004) 24:2585–91. doi:10.1523/JNEUROSCI.4210

03.2004

Ramos VF, Esquenazi A, Villegas MA, Wu T, Hallett M. Temporal discrimination threshold with healthy aging. Neurobiol Aging (2016) 43:174–9. doi:10.1016/ j.neurobiolaging. 2016.04.009

Rocchi L, Casula E, Tocco P, Berardelli A, Rothwell J. Somatosensory temporal discrimination threshold involves inhibitory mechanisms in the primary somatosensory area. J Neurosci (2016) 36:325–35. doi:10.1523/ JNEUROSCI.2008-15.2016

Scontrini A, Conte A, Defazio G, Fiorio M, Fabbrini G, Suppa A, et al. Somatosensory temporal discrimination in patients with primary focal dystonia. J Neurol Neurosurg Psychiatry (2009) 80:1315–9. doi:10.1136/ jnnp.2009.178236
